# Histological, histomorphometric and microtomographic analyses of retrieval hip resurfacing arthroplasty failed at different times

**DOI:** 10.1186/1471-2474-14-47

**Published:** 2013-01-30

**Authors:** Francesca Salamanna, Milena Fini, Annapaola Parrilli, Matteo Cadossi, Nicolò Nicoli Aldini, Gianluca Giavaresi, Deianira Luciani, Sandro Giannini

**Affiliations:** 1 Laboratory of Biocompatibility, Innovative Technologies and Advanced Therapies, Rizzoli Research Innovation Technology, Rizzoli Orthopaedic Institute, Via di Barbiano 1/10, 40136, Bologna, Italy; 2 Laboratory of Preclinical and Surgical Studies, Rizzoli Orthopaedic Institute, Bologna, Italy; 3 II Orthopaedic and Traumatology Clinic, Rizzoli Orthopaedic Institute, Bologna, Italy

**Keywords:** Hip resurfacing arthroplasty, Failure, Histomorphometry, Microtomography

## Abstract

**Background:**

Metal-on-metal hip resurfacing arthroplasty (HR) has been gaining popularity especially for young and active patients. Although different series report good mid-term results, the long-term outcome and failure mechanisms are still concerning. In this consecutive revision case series, 9 retrieved specimens of a failed Birmingham Hip Resurfacing (BHR) were divided according to the time to fracture: 3 specimens failed at less than 6 months (Group 1), 3 failed between 6 months and 3 years (Group 2) and 3 failed later than 3 years (Group 3). The objective of the study was to examine by a specific quantitative histomorphometry and microtomography (micro-CT) method the characteristics of bone quality and its microarchitecture in retrieved metal-on-metal HR.

**Methods:**

A series of 948 BHR were performed between 2001 and 2009. Among these implants 10 failures occurred and nine of these underwent revision surgery and were examined by histomorphometry and micro-CT.

**Results:**

Histomorphometry showed a significant increase in trabecular separation (Tb.Sp) in Group 3 in comparison with Group 1 (113%, p < 0.05). In the top region, micro-CT showed that Groups 2 and 3 presented significant lower bone volume (Group 2: 61%, p < 0.005; Group 3: 1%, p < 0.05), trabecular number (Group 2: 53%, p < 0.005; Group 3: 40%, p < 0.05), and higher Tb.Sp (Group: 71%,p < 0.05) when compared to Group 1. Additionally, histomorphometry showed that the top regions in Group 1 had a significantly lower mean percentage of empty osteocyte lacunae than the top regions in both Group 2 and 3 (p < 0.05).

**Conclusions:**

This study showed that the morphometric parameters considered are crucial for a good understanding of mechanical properties of HR and may be of significant importance in the pathogenesis of HR failure particularly in the development of late fractures.

## Background

Joint replacement is continuously evolving to reduce the invasiveness of surgery, prolong the implant life, decrease complications and improve the patient’s life quality. Resurfacing hip arthroplasty is emerging as an alternative to conventional total hip arthroplasty and has been proposed as an option for the treatment of degenerative hip disease in young, active individuals [1]. HR may present benefits over total hip replacement because femoral bone stock is maintained, there is reduced wear compared with high density polyethylene, it has a large femoral head that could be reduced dislocation rate, it is said to offer the patient increased levels of postoperative activity and is easy to convert into a stemmed prosthesis [2]. The general opinion about this procedure is mainly divided into a favorable one advocated by McMinn et al. [3] and a negative one supported by Spierings et al. [4]. The mid-term results of Birmingham Hip Resurfacing (BHR) suggest a survival rate of about 98% at five years. However, Spierings et al. still consider resurfacing as an experimental design for investigational use only, until long-term follow up confirms its superiority in comparison with total hip replacement [3,4]. Recently various complications, such as femoral neck fracture [5-7], avascular necrosis [8,9] and pseudotumour formation [10,11], as well as unexplained pain, aseptic loosening [12], and osteolysis [4] have been reported. To improve the technique and the success of the treatment, experimental preclinical models can be used to allow the evaluation of biomechanics, biocompatibility, bioactivity and biofunctionality on innovative biomaterials, prosthetic devices and combined therapies. Nevertheless, the retrieval of failed prostheses and the analysis of human implanted devices is one of the most valuable tools to provide information about prostheses that have been submitted to clinical loading and biological and chemical micro-environment during their stay in the body [13]. Indeed, analysis of retrievals can show the histopathological response and the mechanisms of failure [14]. Currently, radiology and histology are the most common procedures to study failed bone implants and some authors have used these techniques to evaluate bone necrosis and fracture risk [6,10,15-18]. Although it is clear that we require a better understanding of the failure mechanisms of the current generation of metal-on metal HR implants, no studies have ever used histomorphometric and microtomographic evaluation to evaluate the characteristics of bone quality and its microarchitecture in retrieved metal-on-metal HR. In fact, from a literature search of the entire MEDLINE database (PubMed research engine) using the MeSH database terms (“hip arthroplasty” [Mesh] OR “hip resurfacing” [Mesh]) AND (“histomorphometric evaluations” [Mesh] OR “x ray microtomography” [Mesh]) no studies were found. Histomorphometry provides information regarding bone tissue and cell dynamics. Similarly, a microtomographic evaluation of the bone structure gives a real estimate of its morphology, especially when it is carried out directly on the entire volume without using predefined volumetric models (plate or rod-model) [19]. Moreover, good correlations were found between the structural parameters determined by microtomography (μCT) images and those assessed on histomorphological slices [20].

Therefore, the main goal of this study was to analyze and examine the characteristics of bone quality and its microarchitecture in retrieved metal-on-metal BHR by a new and specific quantitative histomorphometry and μCT method, never used before. This novel and innovative technique was performed to evaluate whether these 2D and 3D quantitative measurements might be applied to this field of research and give further insight into the failure mechanisms of these implants. This methodology was applied to a small consecutive revision case series taking into account different times to fracture and bone areas located at different distances from the HR dome.

## Methods

### Patient cohort

This is a retrospective observational study in which the protocol was explained to the patients and they gave written informed consent before entering the study (Determination of 20 March 2008, Italian Medicines Agency – AIFA).

A series of 948 (373 female and 575 male) HR (Birmingham Hip Resurfacing, Midland Medical Technologies Ltd, Birmingham, UK: now Smith&Nephew) was performed in our ward at Rizzoli Orthopaedic Institute between early 2001 and late 2009 of whom 941 patients were available for follow-up. Among these implants 10 fractures occurred. Nine of these fractures underwent revision surgery at the Rizzoli Institute and they constitute the series of this study. All these patients underwent HR for primary arthritis of the hip through a postero-lateral approach. The patients’ characteristics of failed HR are reported in Table 1.

**Table 1:** Summary of the cases: patients gender and age (at the time of the primary operation) implant sizes, operation site, time to revision (F: female; M: male)

**Group**	**Time to Revision**	**Age/Gender**	**Implant**	**Notching**	**Cup inclination**	**Stem Neck angle**	**Side**
**Group 1**	3 weeks	70/M	46- mm head −52-mm cup	Present	54°	7°	Left
	2 month	60/F	46-mm head, 52-mm cup	Present	52°	0°	Right
	5 month	50/F	44- mm head −50-mm cup	Present	47°	6°	Right
**Group 2**	14 month	69/F	42- mm head −52-mm cup	Absent	45°	0°	Right
	36 month	47/F	46- mm head −52-mm cup	Absent	46°	0°	Right
	36 month	44/F	42--mm head-48-mm cup	Absent	67°	1°	Right
**Group 3**	4 years	53/M	50- mm head −56-mm cup	Absent	52°	3°	Left
	7 years	50/F	42- mm head −48-mm cup	Absent	60°	4°	Left
	8 years	51/F	46- mm head −52-mm cup	Absent	54°	10°	Left

Fractures were divided into three groups:

- Gross fractures that occurred soon after surgery, earlier than 6 months (Group 1) which presented a pattern involving the implant rim. These fractures were characterized by diffuse reactive changes and varying degrees of perfusion of the proximal bone depending on the vascular injury. It was hypothesized that these aspects may be related to the surgical technique leading to biomechanical changes in the femoral neck by the notch (acute biomechanical fractures);

- Fractures that occurred between 6 months and 3 years (Group 2) and fractures that occurred later than 3 years (Group 3) were defined as late fractures, completely inside the femoral head, with extensive evidence of osteonecrosis. These two groups were divided arbitrarily to highlight the possible presence of a phenomenon that progresses with time. Macroscopically, in each group, necrotic bone tissue appeared pale and white-yellowish with scattered calcifications. One patient of Group 2 experienced a pseudotumor of ileopsoas with an aseptic lymphocytic vasculitis-associated lesions (ALVAL) at 3 years follow up. The acetabular inclination angle was 67°, thus suggesting the presence of edge wear. Metallic debris was evident macroscopically. All patients of Group 3 presented evident macroscopic signs of metallosis without soft tissue involvement, suggesting that osteonecrosis might be also developed by metal corrosion phenomena.

### Surgery

With the patient well secured in the lateral position and under general or spinal anesthesia an extended posterior approach to the hip joint was used in a clean-air operating theatre. The short external rotators were released, the gluteus maximus was detached from its insertion at the linea aspera, and a circumferential capsulotomy was performed. The femoral head was dislocated anteriorly and the acetabulum reamed sequentially. Peripheral acetabular osteophytes were excised and a trial component which was 1 mm smaller than the intended final implant was used to confirm that a tight fit had been obtained. If this fixation was satisfactory, the definitive acetabular component was then impacted. Standard instrumentation was used to align and position the guide rod for the preparation of the femoral head using the lateral cortical pin and out-rigger. The head was reamed to house a femoral component that matched the implanted acetabular component. The femoral implant was positioned and secured with Simplex (Howmedica International, Limerick, Ireland) low viscosity cement. The hip was then reduced and the short external rotators and gluteus maximus tendon repaired.

### Histological and histomorphometric analyses

**Figure 1:**
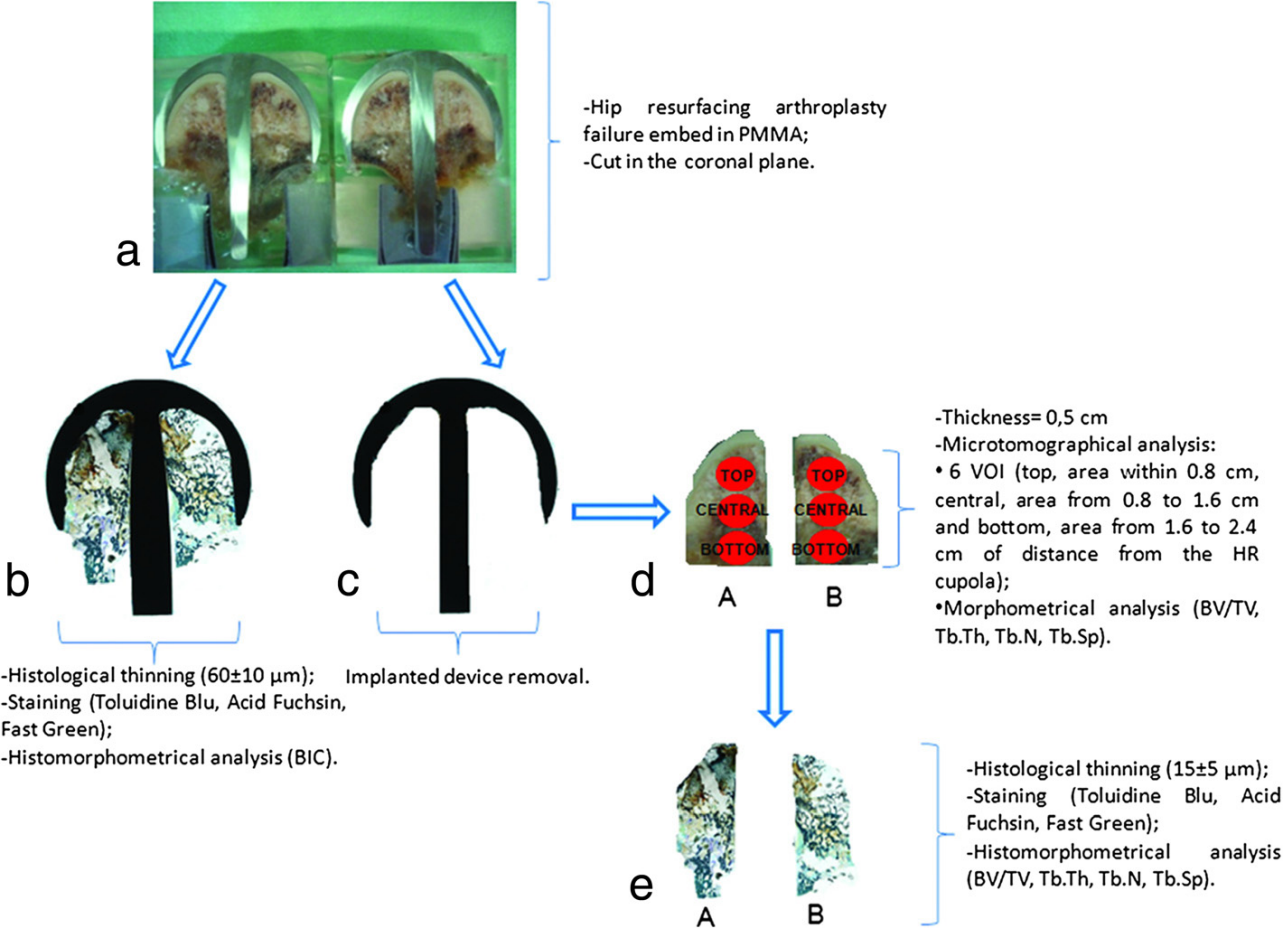
**Schematic representation of the methodology for sample analyses.** Epson 2480 Scanner, 600dpi of resolution. **a**) The specimens were embedded in PMMA and cut along the coronal plane, **b**) sections containing the implants were used for histology and histomorphometric measurement (BIC), **c**) after the removal of the prosthesis, **d**) two bone compartments (A and B) were used for μCT, **e**) thinned and processed for routine histological and histomorphometric analyses.

At revision, the femoral component together with the femoral head and neck bone was resected *en bloc* and immediately placed in buffered 4% paraformaldehyde. No acetabular components were removed. After fixation, the bone-metal composite specimens were dehydrated by placing them in graded series of increasing percentage of alcohol with one step in 50% alcohol, one step in 75% alcohol, two steps in 95% alcohol, and two steps in 100% alcohol, for 48 hrs per each step. After dehydration, the undecalcified specimens were infiltrated by exposing them to methyl methacrylate solution (Merck, Germany). The infiltration was completed with use of methyl methacrylate combined with benzoyl peroxide (Sigma-Aldrich) and poly-methyl-methacrylate (PMMA) (Sigma-Aldrich) under *vacuum* condition for four days. The different steps of the study are summarized in Figure 1. The embedded specimens were cut in middle along the coronal plane by a saw with a diamond-coated band (EXAKT, GmbH & Co., Norderstedt, Germany). The orientation of the specimens during the cutting process was preserved, thus maintaining a low feed force coupled with the automatic control of the cutting band. One section of about 0.5 cm thick and two sections of 350 ± 100 μm thick were obtained for each HR sample. The implanted device was removed from the first section and the section was analyzed by μCT as described below. Then, it was automatically thinned (EXAKT Systems) to 15 ± 5 μm with different abrasive papers (EXAKT Abrasive Disc), from 80 to 2000 grit in steps of 15 minutes each and used for histomorphometric measurements. By the same procedure the other two sections of HR retrieved were automatically thinned to 60 ± 10 μm and used to measure the contact between bone and prosthetic stem. Next, the sections were stained with Toluidine Blue, Acid Fuchsin, Fast Green, and processed for routine histological analyses. Histological analyses were performed by using a transmission and polarized light AxioSkop Microscope (Carl Zeiss GmbH, Germany) at a magnification from 1.25x to 20x. After prosthesis removal, two different compartments of the femoral head were considered: A lateral and B medial. Each compartment was split into 3 regions of interest (ROI) depending on the distance from the HR dome: within 0.8 cm (top), from 0.8 to 1.6 cm (central) and from 1.6 to 2.4 cm (bottom). Finally, histomorphometric analyses were carried out with computerized image analysis Axio-Vision-4.6 (Carl Zeiss). Bone histomorphometry measurements were taken semi-automatically at a magnification of 1.25x by two experienced blinded investigators, by dividing the sections of compartments A and B into different quadrants. The bone-to-implant-contact (BIC) was measured at the interface between bone and prosthesis stem as the rate between the stem surface directly in contact with bone without the interposition of fibrous tissue/the total interface length x 100 (%). The bone histomorphometric parameters were measured in accordance with the *Histomorphometry Nomenclature by the Committee of the American Society for Bone and Mineral Research*[21]:

– Bone Volume (BV/TV,%): the whole spongy bone area, expressed as a percentage of the total tissue area in the sampling site and converted to a volume;

– Trabecular Number (Tb.N, mm^-1^): index of density of trabeculae;

– Trabecular Thickness (Tb.Th, μm): index of the width of trabeculae;

–Trabecular Separation (Tb.Sp, μm): index of the distance of trabeculae;

– Cement Thickness (Cm.Th): index of the width of the cement on the dome surface.

For each sample, the mean percentage of empty lacunae in five regions of interest in the top, central and bottom part, at a magnification of 20x, was determined by two experienced blinded investigators using the method of Steffen et al. [22].

### Microtomographic analysis

As shown in Figure 1, μCT assessment was carried out on a 0.5-cm-thick embedded section of the samples using the Skyscan 1172 computed microtomographic system (Kontich, Belgium). The scans were performed with a 100 kV voltage source and 100 μA current source. Images were acquired with a pixel size of 12 μm, an aluminum filter 0.5 mm, and a sample rotation step of 180° and 0.4°. The scans were later reconstructed to obtain microtomographic sections. Reconstruction was performed by using NRecon (v1.6.2.0) software: a specific alignment was used for each sample and a medium intensity ring artifact correction was applied. Microtomographic 3D analyses were performed using CTAn (v.1.10.1.3) software and, as for histologic evaluation, considering 3 Volumes of interest (VOI) in each compartment (A and B): at the top, within 0.8 cm from the HR dome, in the centre, from 0.8 to 1.6 cm from the HR dome, and at the bottom, 1.6 to 2.4 cm from the HR dome. The morphometric parameters considered were derived in part from those already defined by Parfitt [21]:

Bone volume density (BV/TV,%), expressed as a ratio between the volume of bone measured in the VOI and the total volume of the considered VOI;

– Trabecular thickness (TbTh, μm) measured as a true model-independent 3D value;

– Trabecular separation (TbSp, μm), derived from the volume-based local thickness just applying the method to the space between trabeculae;

– Trabecular number (TbN,mm^-1^), defined as: 1/(*TbTh + TbSp*)

### Statistical analysis

Statistical analysis was performed by using the SPSS Inc v.12 software. Data were reported as Mean ± SD at a significant level of p < 0.05. After checking normal distribution (Shapiro-Wilks test) and homogeneity of variance (Levene test), the non-parametric Kruskall-Wallis test followed by Mann–Whitney test with Monte Carlo methods to compute probability were carried out to compare histomorphometric results among groups.

## Results

### Histological results

#### Group 1

**Figure 2:**
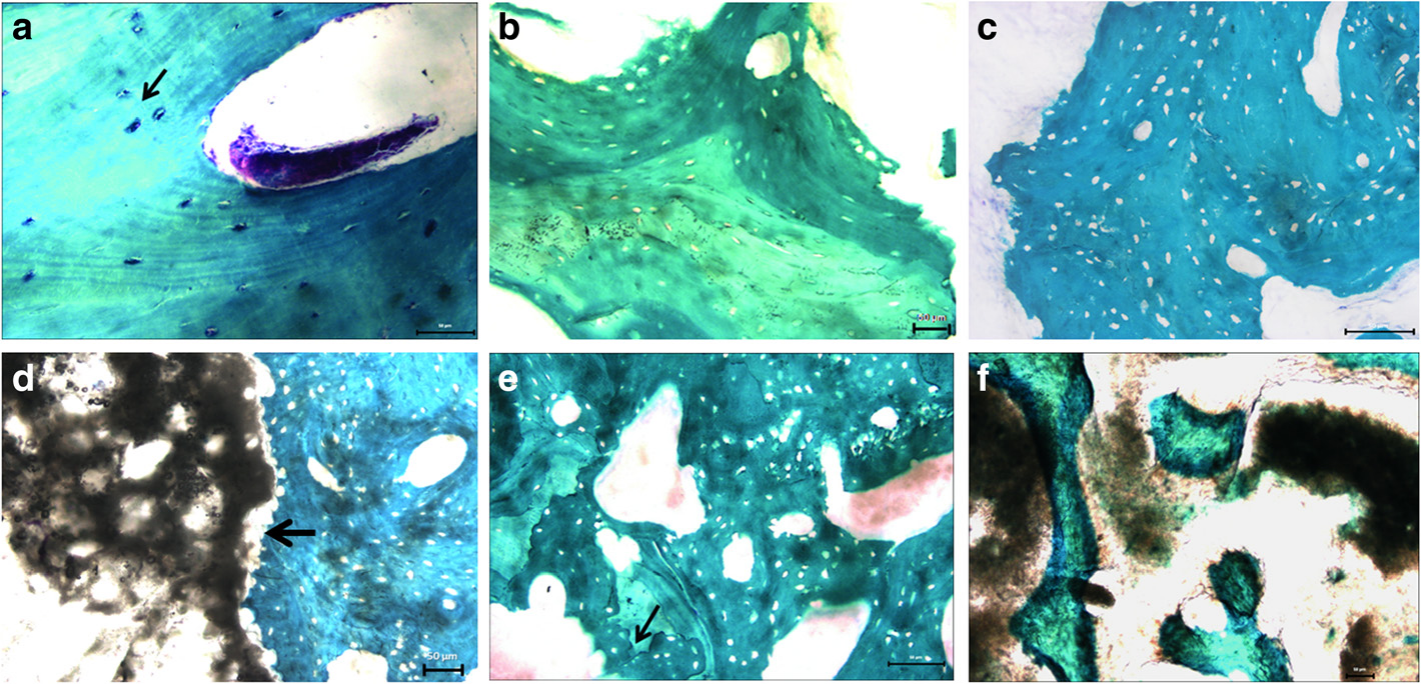
**Histology of specimens revised in Group 1 (a, b), Group 2 (c, d) and Group 3 (e, f); sections are representative of bone tissue at increasing distances from the HR dome: within 0.8 cm (top) (a, c, e), from 1.6 to 2.4 cm (b, d, f) (bottom).** Toluidine Blue, Acid Fuchsin, Fast Green staining. **a**) trabecular lamellar bone with evident evenly-spread osteocytes, orientated with the longest axis in the direction of the lamellae contained in the bone lacunae (arrows), resolution 20x; **b**) necrotic bone tissue, resolution 10x; **c**) necrotic tissue, resolution 20x; **d**) necrotic bone tissue infiltrated with aggregates of small dark metal wear-debris particles (metallosis) (arrows), resolution 10x; **e**) loss of normal trabecular bone microarchitecture, uneven edges due to the resorption of necrotic bone (arrows) resolution 20x; **f**) metallosis in close association with the necrotic bone trabeculae, resolution 10x.

The mean time-to-revision was 10.3 ± 8.7 weeks. No difference was found by histological analysis in this group (Figure 2). A thin layer of cement was present at the bone-dome interface and intraosseous cement penetration was recognized by its hard consistency, fine granular structure, and color in the top regions. Focal areas of osteonecrosis with trabecular lamellar bone with empty lacunae were observed in the central and bottom regions (Figure 2a,b). In the bone areas adjacent to the HR dome (top ROI) normal bone microarchitecture and morphology with osteocyte nuclei in the lacunae were observed (Figure 2a). In the 5-month case the areas of focal osteonecrosis with bone trabeculae without stainable osteocytes were associated with signs of appositional new bone formation.

#### Group 2

The mean time–to-revision was 28.6 ± 12.7 months. No differences were observed among the 3 analyzed prostheses that had failed from 14 to 36 months (Figure 2c,d). A cement mantle was present at the dome and intraosseous cement penetration was observed in the top region and in a small part of the central region. An absence of osteocyte nuclei within bone lacunae was observed in all cases in all ROIs. Thickened cancellous bone trabeculae were sometimes observed with extensive formation of appositional new bone on the surface of necrotic trabeculae (Figure 2c). Signs of metallosis with infiltration and accumulation of metallic wear debris inside the periprosthetic structures were clearly visible in the two patients that failed at longer follow up times (Figure 2d). At 36 months a considerable amount of connective tissue was observed.

#### Group 3

The mean time-to-revision was 6.3 ± 2.1 years. Sectioning of the implant revealed a thin layer of cement at the dome and a penetration of cement deep into the bone was observed in the top and central region. Femoral head section analyses showed a decrease in bone mass with partial necrosis in each examined sample. Histological examination confirmed the presence of a massive metallosis revealing granulomatous tissue with extensive pigmented deposits in all examined cases, which was more evident at 7 and 8 years with bone rarefaction present in all ROIs of the femoral head (Figure 2e,f).

### Histomorphometric results

**Figure 3:**
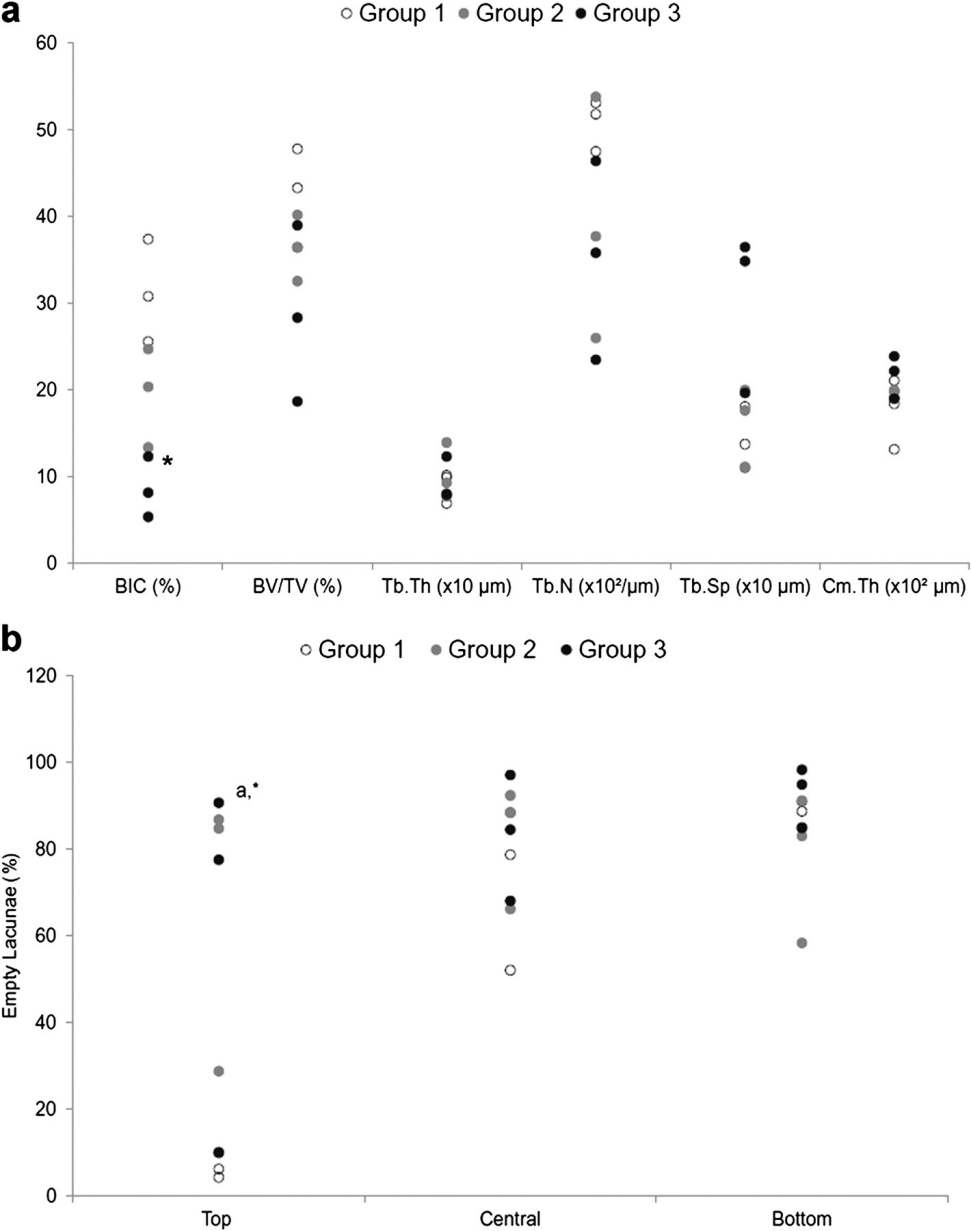
**Dot plot of (a) histomorphometric parameters and (b) percentage of empty osteocyte lacunae in the different ROIs (top, central, bottom) for each Group.** Mann–Whitney test: (**a**) Group 3 versus Group 1 (*, p < 0.05); (**b**) ^a^, Top region versus central and bottom regions (p < 0.05).

The 2D histomorphometric tests were performed on the sections where the implanted device was present for BIC and Cm.Th measurement and in those where the HR had been removed for BV/TV, Tb.Th, Tb.N, Tb.Sp and percentage of empty lacunae measurements. The results of histomorphometric parameters are reported in Figure 3a and b. The Kruskal-Wallis test highlighted statistically significant differences between groups for BIC (*p* = 0.004). The Mann–Whitney test showed that Group 3 presented statistically significantly lower BIC values (71%, *p* = 0.004) when compared to those of Group 1. The results of the percentage of empty lacunae obtained from each separate ROI (top, middle and bottom) are summarized in Figure 3b. Statistically significant differences were found for empty lacunae in Group 1 (*p* = 0.05) between the top region and the others. Additionally, the top regions in Group 1 had a significantly lower mean percentage of empty osteocyte lacunae than top regions in both Group 2 and Group 3 (*p* = 0.05).

### Microtomographic results

**Figure 4:**
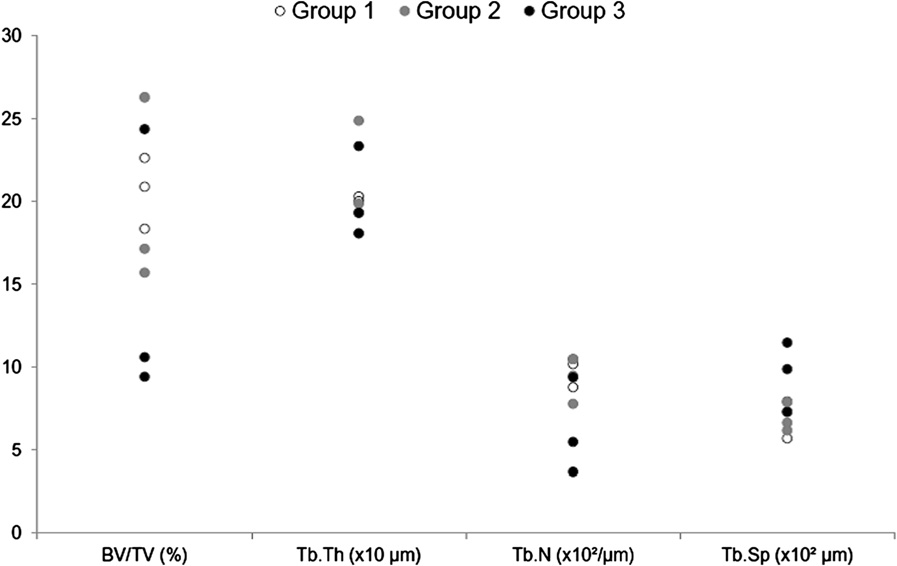
Dot plot of microtomographic analysis for each Group.

**Figure 5:**
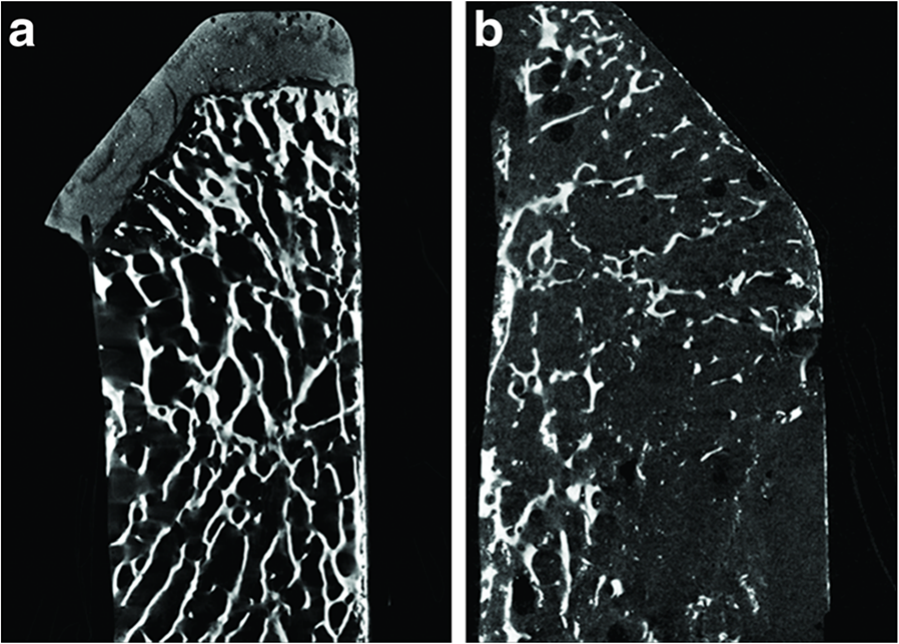
**Microtomographic sections of HR failure after prostheses removal.a)** Group 1 (5 months); **b)** Group 3 (7 years) showing an important bone rarefaction.

**Figure 6:**
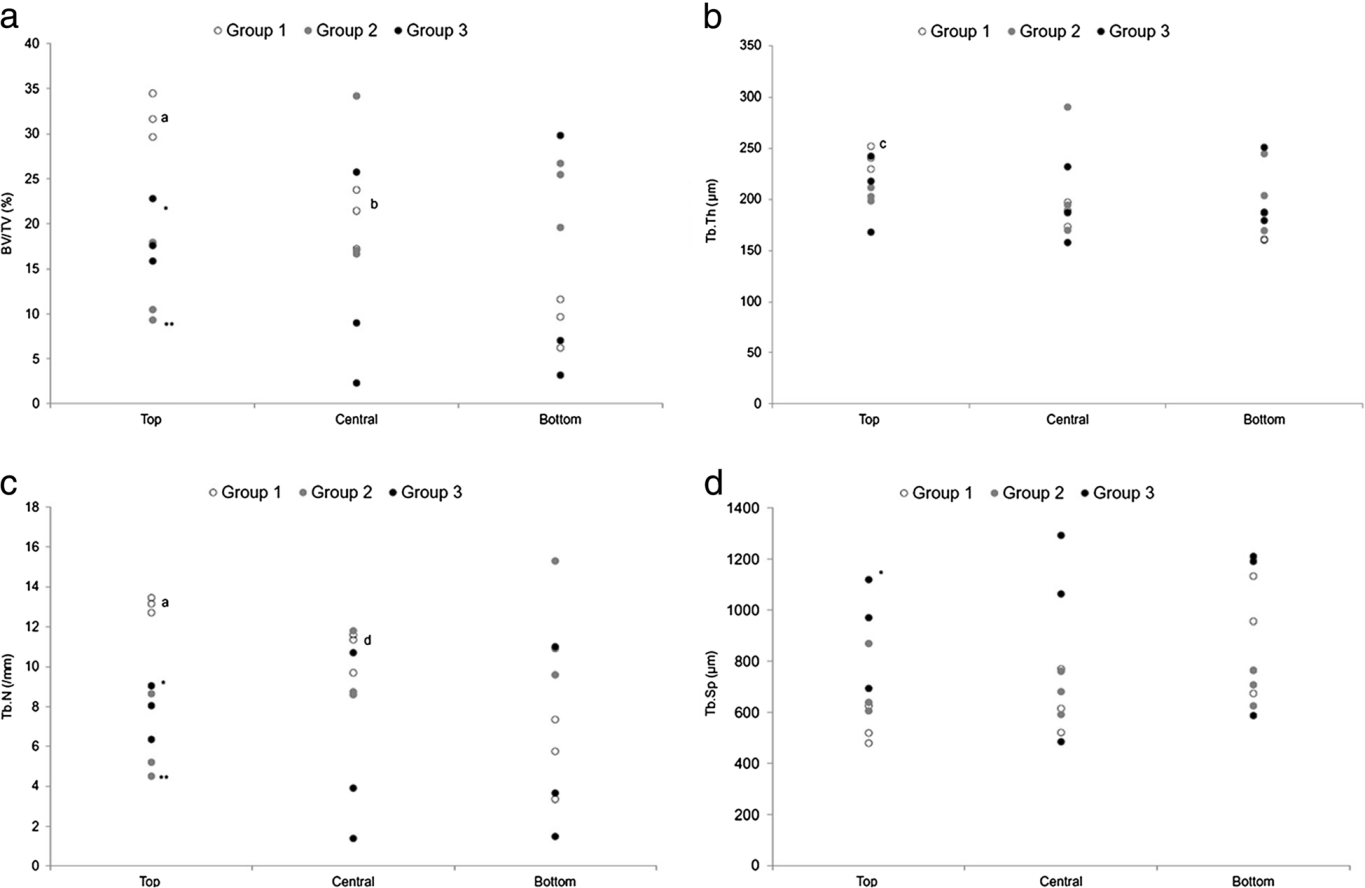
**Dot plot of microtomographic data split into the different ROIs (top, central, bottom) (a) BV/TV; (b) Tb.Th; (c) Tb.N; (d) Tb.Sp.** Mann–Whitney test: - comparison between terms: medium Group 2 and long-term Group 3 groups versus short-term group Group 1 (*, *p <* 0.05; **, *p <* 0.005); -^a^, Top region versus bottom region (*p <* 0.005); ^b^, Central region versus top and bottom regions (*p <* 0.05); ^c^, Top region versus central and bottom regions (*p <* 0.05); ^d^, Central region versus bottom region (*p* < 0.05).

The 3D microtomographic analysis was carried out on the sections after the removal of the implanted device. The results of μCT parameters for each patient are reported in Figure 4. Data are in parallel with those of histomorphometric results and showed that bone rarefaction (as measured by BV/TV, Tb.N, Tb.Sp) progressively changes over time (Figure 5a,b). The comparison between Group 1 and Group 3 showed that BV/TV and Tb.N decreased by about 28% and 37%, respectively, whereas Tb.Sp increased by about 37%, but the differences did not reach statistical significance. Microtomographic data obtained from each separate ROI (top, middle and bottom) are reported in Figure 6a-d. Statistically significant differences between Groups in the top ROI were found for BV/TV (*p* = 0.025), Tb.N (*p* = 0.025) and Tb.Sp (*p* = 0.028). In the top ROI, the Mann–Whitney test showed that Group 2 and Group 3 presented significantly lower BV/TV (Group 2: 61%, *p* = 0.05; Group 3: 41%, *p* = 0.05) and Tb.N (Group 2: 53%, *p* = 0.05; Group 3: 40%, *p* = 0.05), and higher Tb.Sp (Group 3: 71%, *p* = 0.05) when compared to those of Group 1.

## Discussion

The main goal of this study was to evaluate the characteristics of bone quality and its microarchitecture in a series of femoral heads that failed at different times for different reasons by adopting an innovative and specific quantitative histomorphometry and μCT methodology. To do this nine failures were considered, which were split into groups depending on the failure time: 3 specimens failed at less than 6 months (Group 1), 3 failed between 6 months and 3 years (Group 2) and 3 failed at more than 3 years (Group 3) after HR surgery. In comparison with other studies, in this one the Groups were divided arbitrarily to highlight the possible presence of a phenomenon that progresses over time.

Histological evaluation showed the presence of focal areas of osteonecrosis with empty lacunae in the Group 1. In Group 2 and Group 3 partial osteonecrosis also was present; nevertheless, newly formed bone was visible on the surface of the necrotic bone trabeculae. These data were in agreement with those of Steffen et al. who showed that the necrotic changes were associated with appositional new bone formation and marrow fibrosis [14]. In fact, proliferating cells spread through the narrow spaces between the dead trabeculae, differentiate into osteoblast, and subsequently form appositional new bone on the surface of dead trabeculae. At the same time, they initiate osteoclastic resorption of necrotic bone. Osteoclastic resorption, modulated by cytokines released from osteoblast, is crucial for the balance of the repair processes. The bone may be markedly weakened if resorption occurs at the interface of the viable and dead bone, or if revascularization and new bone formation in necrotic areas is prevented by the formation of a fibrous scar [22]. Bone atrophy was observed at histological analyses only in Groups 2 and 3 and these results were confirmed by micro-CT (BV/TV, Tb.N, Tb.Sp) thus suggesting a possible role of mechanical factors (stress shielding). Metallosis, with infiltration and accumulation of metallic wear debris, was visible in Group 2 and 3. Therefore, as shown by other authors who studied the failure mechanism of HR prostheses by conventional radiography and qualitative histology, the present histological analyses confirmed that aseptic necrosis and bone rarefaction might play a crucial role in late failures of HR [6,14,15,17,23-25].

Unlike previous studies, the present one took into consideration 3 groups of patients according to failure times (from 3 weeks to 8 years); quantitative measurements were performed with histomorphometry and μCT and 3 peri-implant bone regions at different distances from the HR dome (within 0.8 cm (top), from 0.8 to 1.6 cm (central) and from 1.6 to 2.4 cm (bottom)) were considered. This was possible through resin embedding of the femoral heads containing the prostheses, cutting along the coronal plane of the macro-sections and subsequent removal by pressure of the prosthesis that permitted the accurate evaluation of bone histology and microarchitecture with both 2D (histomorphometry) and 3D (μCT) techniques. A different bone architecture was highlighted within each group and, in particular, between the Group 1 and Group 3. Both 2D and 3D measurements showed that bone density decreases over time especially in Group 3 if compared with Group 2 and Group 1. 3D data of different ROIs (top, central, bottom) of both lateral and medial compartments showed a significant decrease in bone quality over time in the top ROI near the dome. This was confirmed by the significant differences in BV/TV, Tb.N and Tb.Sp between Group 1 versus Group 2 and Group 3 in the top ROI. This tendency was visible also in the lower ROIs but bone values did not reach statistical significance.

In the present study bone resorption was observed within the resurfaced femoral head and around the proximal part of the stem. Whereas bone remodeling is a feature of normal metabolism in healthy and osteoarthritic bone, the BHR may result in stress shielding with consequent resorption and narrowing of the femoral neck due to altered loading conditions. This stress shielding is probably due to the implant design with long-stems. In fact, Bidyut Pal and coworkers showed that bone resorption was considerably less for short-stem designs; the short-stem design having stem-bone contact not only led to a more physiological stress distribution but also to bone apposition in the superior side of the resurfaced head [26]. Moreover, 2D results showed significant differences also in the percentage of empty lacunae between Group 1 versus Group 2 and Group 3 in the top ROI. The proportion of empty lacunae gradually increased over the time after surgery.

The present results were in agreement with those of Steffen et al. who showed that samples from late fractures had a significantly higher proportion (84%) of empty osteocyte lacunae within the trabecular bone compared with those of samples from fractures occurring within the first month (48%) after HR [14]. Moreover, in the present study the higher mean percentage of empty lacunae in the central and bottom regions of Group 1 was probably due to a vascular injury. This controversial result might be explained by analyzing the surgical technique. During femoral head preparation the top region is always removed, thus eliminating the bone volume more subjected to osteonecrosis. Moreover, the residual blood supplied comes from the lateral femoral circumflex artery and a recent report [27] shows two more possible sources of blood supply to the femoral head. These two vessels were identified as the anterior nutrient artery of the femoral neck which origins from the lateral femoral circumflex artery and the inferior branch of the deep branch of the superior gluteal artery.

In the present series all the operations were performed through a posterior approach which is known to disrupt the medial circumflex artery; nevertheless, the failure rate due to bone necrosis was low. Similar findings were observed by Mcbride et al. who reported the same implant survival regardless of surgical approach [28]. By using a surgical approach that preserves the blood supply it might be possible to obtain an improved implant survival at longer follow up [14,19,29].

The BIC measurement should not be considered as an index of osteointegration because the surgical procedure of HR insertion is not aimed at achieving primary fixation between the bone and the stem as for traditional arthroplasties. However, a progressive decrease of bone in contact around the stem was observed and the difference was significant between Group 1 and Group 3 patients. The decrease in BIC was probably due to the bone rarefaction, which involves the femoral head; it remains to be seen whether it might also be related to a progressive prosthesis loosening over time. In the present study histological and microtomographic analyses suggest that both processes, bone rarefaction and osteonecrosis, start from the bottom of the peri-implant bone and reach the top region adjacent to the HR dome in the Group 2 and 3. Osteonecrosis is expected to start from the top ROI which is far away from blood vessels and probably more influenced by the presence of cement but the findings in the present study showed the contrary. In fact, some sort of stress shielding due to its close relationship with the implant might be the true reason for this particular finding.

The current study has several limitations. First of all the small number of cases prevents any solid conclusions to be drawn about the real failure mechanisms of HR and the progression of femoral head damage. The inter-individual variability between patients and osseous changes should also be taken into account. Nevertheless, it was not the primary objective of this study to define the pathophysiology of HR prosthesis failure. To the present authors’ knowledge, quantitative methodologies for measuring bone quality and its microarchitecture have never been used to study retrieved HR prostheses. In the present study the histomorphometric and microtomographic evaluations allowed bone microarchitecture alterations to be quantified.

## Conclusions

The objective of the study was to examine the characteristics of bone quality and its microarchitecture in retrieved metal-on-metal HR by a specific quantitative histomorphometry and μCT method. The results showed that the morphometric parameters considered were crucial for a good understanding of the mechanical properties of HR and may be of significant and essential importance in the pathogenesis of HR failure particularly in the development of late fractures. Although there are several good reports on the survival rate of HR at mid-term follow up, the biological changes of the femoral head underlying the implant over time should always be considered. It remains to be seen whether other late failures will occur. HR is still a good indication for young and active patients; nevertheless good bone quality remains the crucial element to support the implant at longer follow-up.

## Abbreviations

HR: Resurfacing Arthroplasty; BHR: Birmingham Hip Resurfacing; μCT: Microtomography; BV/TV: Bone Volume; Tb.N: Trabecular Number; Tb.Th: Trabecular Thickness; Tb.Sp: Trabecular Separation; Cm.Th: Cement Thickness.

## Competing interests

The authors declare that they have no competing of interests.

## Authors’ contributions

FS provided input on the interpretation of the results and drafted the manuscript; MF participated in designing the study and helped edit the manuscript; AP carried out the microcomputer tomography analysis; MC participated in discussions of the project and revised it critically for important intellectual content; NNA provided input on the interpretation of the results; GG participated in designing the study and carried out the statistical analysis; DL provided input on the interpretation of the results; SG designed the study, provided input on the interpretation of the results and helped to draft the manuscript. All authors read and approved the final manuscript.

## Pre-publication history

The pre-publication history for this paper can be accessed here:

http://www.biomedcentral.com/1471-2474/14/47/prepub
